# Outcome of Atezolizumab Plus Bevacizumab Combination Therapy in High-Risk Patients with Advanced Hepatocellular Carcinoma

**DOI:** 10.3390/cancers16040838

**Published:** 2024-02-19

**Authors:** Sang Youn Hwang, Hyun Young Woo, Jeong Heo, Hyung Jun Kim, Young Joo Park, Ki Youn Yi, Yu Rim Lee, Soo Young Park, Woo Jin Chung, Byoung Kuk Jang, Won Young Tak

**Affiliations:** 1 Department of Internal Medicine, Dongnam Institute of Radiologic & Medical Sciences, Busan 46033, Republic of Korea; mongmani@daum.net (S.Y.H.); phorni@naver.com (H.J.K.); 2 Department of Internal Medicine, College of Medicine, Pusan National University and Biomedical Research Institute, Pusan National University Hospital, Busan 49241, Republic of Korea; who54@hanmail.net (H.Y.W.); juya0630@naver.com (Y.J.P.); yikiyoun@hanmail.net (K.Y.Y.); 3 Department of Internal Medicine, School of Medicine, Kyungpook National University, Kyungpook National University Hospital, Daegu 41944, Republic of Korea; deblue00@naver.com (Y.R.L.); psyoung0419@gmail.com (S.Y.P.); 4 Department of Internal Medicine, Keimyung University School of Medicine, Daegu 42601, Republic of Korea; chung50@dsmc.or.kr (W.J.C.); jangha106@dsmc.or.kr (B.K.J.)

**Keywords:** hepatocellular carcinoma, atezolizumab, bevacizumab, radiation

## Abstract

**Simple Summary:**

Although ATE + BEV treatment provides improved therapeutic efficacy, in our study, high-risk populations such as patients with grade Vp4 portal vein thrombus, bile duct invasion, or more than 50% liver infiltration had poor responses. This study aimed to investigate real-world outcomes and prognostic factors in high-risk patients with advanced HCC treated with atezolizumab plus bevacizumab. Unlike what was reported in the IMbrave150 study, atezolizumab plus bevacizumab treatment had consistent efficacy and tolerability in both the total and high-risk population in our study. Radiation therapy combined with atezolizumab plus bevacizumab treatment might be helpful to improve progression-free survival and overall survival in high-risk groups.

**Abstract:**

Real-world data regarding treatment with atezolizumab plus bevacizumab in high-risk patients with advanced HCC are lacking. In this multicenter retrospective cohort study, a total of 215 patients with advanced HCC received atezolizumab plus bevacizumab treatment at four tertiary hospitals. High-risk patients were those with grade Vp4 portal vein thrombus, bile duct invasion, or more than 50% liver infiltration. In total, 98 (45.6%) were the high-risk population, 186 (86.5%) were considered to be Child–Pugh class A, and 128 (59.5%) had previously received neoadjuvant or concomitant radiation treatment. Median overall survival (OS) was 11.25 months (95% CI, 9.50–13.10), and the median progression-free survival (PFS) was 8.00 months (95% CI, 6.82–9.18). In the high-risk population, the median OS was 10 months (95% CI, 8.19–11.82) and the median PFS was 6.50 months (95% CI, 3.93–9.08). In the high-risk population, multivariate analysis indicated that radiation therapy and lower ALBI grade were associated with better OS and PFS. A total of 177 (82.3%) patients experienced adverse events of any grade, the most common being proteinuria (23.7%). Atezolizumab plus bevacizumab treatment showed consistent efficacy and tolerability in both the total and high-risk population. Radiation therapy combined with atezolizumab plus bevacizumab treatment might be helpful to improve PFS and OS in high-risk populations.

## 1. Introduction

Hepatocellular carcinoma (HCC) is the most common type of cancer of the hepatobiliary tract and the third leading cause of cancer-related deaths worldwide [1]. Because cancer-related symptoms usually appear after the progression of HCC to an advanced stage, most of these patients have unresectable cancer upon diagnosis and have a poor prognosis [2]. Before 2020, sorafenib and lenvatinib were proved to be effective in the first-line systemic treatment of HCC on the basis of survival benefits in phase III randomized controlled trials (RCTs). Sorafenib showed better median overall survival (OS) compared to placebo (10.7 vs. 7.9 months) in the SHARP trial [3], and lenvatinib proved to have non-inferior median OS compared to sorafenib (13.6 vs. 12.7 months) in the REFLECT trial [4]. Recently, some RCTs have reported that immune checkpoint inhibitor (ICI)-based combination treatment has better median OS compared to sorafenib monotherapy. In the HIMALAYA trial, durvalumab–tremelimumab combination therapy demonstrated improved OS (16.4 vs. 13.8 months) compared to sorafenib [5]. The 2020 IMbrave150 trial showed that treatment with atezolizumab plus bevacizumab (ATE + BEV), rather than sorafenib alone, led to significantly improved OS (19.2 vs. 13.4 months) and progression-free survival (PFS, 6.9 vs. 4.3 months) [6,7]. Although adverse events such as gastrointestinal bleeding, proteinuria, and hypertension related with bevacizumab have to be considered because they have been reported in a significant percentage in some studies, including IMbrave150, it was concluded that these adverse events are manageable [8]. Therefore, this ICI-based combination therapy is now approved worldwide as a first-line treatment for unresectable HCC [6,7], and the Barcelona Clinic Liver Cancer (BCLC) group guidelines recommended that ICI-based treatment is considered preferentially as a first-line systemic treatment of HCC if there are no contraindications in patients, such as an autoimmune disease or organ transplantation [9]. In particular, real-world data are already available on ATE + BEV therapy because it was used earlier on a global scale after it received FDA approval compared to durvalumab–tremelimumab therapy. According to several recent studies with a small amount of patients receiving ATE + BEV therapy, overall response rate (ORR) and disease control rate (DCR) have been reported in the range of 28–51% and 66.1–89.5%, and median OS and PFS have been shown to be in the range of 12–22.2 months and 5.2–8 months [10,11,12,13]. Gao et al. performed a single-arm meta-analysis focused on the efficacy and safety of ATE + BEV in 23 studies, which included 3168 patients, and reported that the ORR was 26%, and median OS and PFS were 14.2 months and 6.66 months [14].

Although ATE + BEV treatment provides improved therapeutic efficacy, a substantial number of these patients (high-risk population) have had poor responses. This high-risk group consisted of patients with grade Vp4 portal vein thrombus, bile duct invasion, or more than 50% liver infiltration [7]. The IMbrave150 study focused on ATE + BEV reported that this high-risk group had a median OS of 7.6 months, significantly longer than the sorafenib group (5.5 months), but much shorter than the total population (19.2 months) and the population who were not at high risk (22.8 months) [15]. However, no studies have been conducted on these high-risk patients in real-world settings. 

Therefore, we performed a retrospective cohort study to evaluate the real-world efficacy and safety of ATE + BEV in patients with advanced HCC, some of whom were suffering from high-risk cancer.

## 2. Material and Methods

This multicenter, retrospective cohort study evaluated the real-world efficacy and safety of ATE + BEV in patients with advanced HCC. From 1 January 2020 to 30 June 2022, 215 patients with advanced HCC who received ATE + BEV therapy at four different university hospitals were evaluated. Advanced HCC was defined as locally advanced, metastatic, or unresectable hepatocellular carcinoma (or both) [6]. HCC was determined by histological or clinical examination according to the current HCC guidelines of the American Association for the Study of Liver Diseases [16]. Patients were eligible if they had not previously received systemic therapy for HCC, had measurable disease that was unamenable to curative or locoregional therapies, or experienced cancer progression after previous locoregional therapy. Patients were allocated the status of being high-risk if they met one of the following: grade Vp4 portal vein thrombus, bile duct invasion, or >50% liver infiltration [15]. This study was reviewed and approved by the Institutional Review Board, Republic of Korea (IRB study No. 2301-012-123), and was performed in accordance with the Declaration of Helsinki. The Institutional Review Board waived the requirement for informed consent because of the retrospective nature of this study and because all patient data were anonymized. All data were accessed for research purposes from the date of IRB approval (30 January 2023) to 5 July 2023.

We evaluated patients’ HCC stage before the first dose of ATE + BEV therapy was administered using triple-phase computed tomography (CT) or magnetic resonance imaging (MRI) of the liver, CT of the lungs, and bone scintigraphy. Prophylactic endoscopy was performed to identify varices and ulcerations. Before receiving the first dose of ATE + BEV therapy, patients with gastric or duodenal ulcerations received proton pump inhibitors, and patients with high-risk varices received non-selective beta-blockers. Ascites was diagnosed via a physical examination and radiology and was managed with diuretics at the discretion of the physicians at each institution.

Clinical data, including age, sex, Eastern Cooperative Oncology Group performance status, alcohol consumption, and previous treatments (resection, transarterial chemoembolization [TACE], radiofrequency ablation [RFA], and radiation), were recorded. Radiation therapy was performed before or concomitantly with ATE + BEV. Radiation was considered for the treatment of intrahepatic HCC with incomplete response to TACE, portal venous tumor thrombus, or symptomatic extrahepatic metastasis such as bone, lymph node, or adrenal gland for the purpose of symptomatic palliation, recurrent, or refractory HCC after locoregional therapy following the guidelines of the Korean Liver Cancer Association [17]. Radiation therapy was recommended following multidisciplinary evaluation and performed when patients agreed to receive radiation therapy. In this study, radiation therapy was performed as neoadjuvant and concomitant, and the meanings of each are as follows: Neoadjuvant radiation therapy means that radiotherapy was administered and completed immediately before the combination treatment of atezolizumab and bevacizumab. Concomitant radiation therapy refers to radiation therapy administered during combined treatment with atezolizumab and bevacizumab. Blood data, including markers for hepatitis B and hepatitis C viruses, complete blood count, Child–Pugh score, albumin–bilirubin (ALBI) grade, alpha-fetoprotein (AFP), and des-gamma-carboxy prothrombin (DCP), were also evaluated. 

The dose and schedule of intravenous ATE + BEV were 1200 mg of ATE and 15 mg/kg body weight of BEV every 3 weeks. Dose modification was not performed, and treatment interruption was performed as described in the protocol of the IMbrave 150 trial [6]. ATE + BEV treatment was continued until there was an unacceptable toxic effect, loss of clinical benefit, or patient refusal. A patient was allowed to continue treatment after disease progression if the clinician found evidence of clinical benefit and if there were no definitive symptoms or signs of unequivocal disease progression. 

Treatment efficacy was evaluated based on OS, PFS, and tumor response. The tumor response was evaluated every 3–4 cycles (9–12 weeks). Treatment response was evaluated using the Response Evaluation Criteria in Solid Tumors version 1.1 [18], and imaging results from CT and/or MRI were used to identify complete response (CR), partial response (PR), stable disease (SD), and progressive disease (PD). The ORR was defined as the percentage of patients who achieved CR or PR, and the DCR was defined as the percentage of patients who achieved CR, PR, or SD.

Safety was continuously evaluated by assessing the incidence and severity of adverse events (AEs) according to the National Cancer Institute Common Terminology Criteria for Adverse Events version 5.0. Efficacy and safety were evaluated in all patients who received at least one dose of ATE + BEV.

### Statistical Analysis

Data for all variables are expressed as mean ± standard deviation, median (range), or number (percentage). Differences in continuous variables were assessed using Student’s *t*-test or the Mann–Whitney test, and differences in categorical variables were assessed using the chi-squared test or Fisher’s exact test. OS was defined as the time from the initiation of the ATE + BEV regimen to death or the last follow-up, and PFS was defined as the time from the initiation of the ATE + BEV regimen to PD or death. Kaplan–Meier survival curves were plotted for the different groups and compared using the log-rank test. Missing data or those lost to follow-up were considered PD or death. A multivariable Cox proportional hazards regression model was used to identify independent predictors of OS and PFS. Multivariate analysis was performed with factors with a *p*-value of less than 0.05 in univariate analysis. All statistical analyses were performed using SPSS version 25.0 (IBM Corp., Armonk, NY, USA). For all comparisons, a *p*-value < 0.05 was considered significant.

## 3. Results

### 3.1. Patient Characteristics

We retrospectively examined the records of 215 patients with advanced HCC who received ATE + BEV treatment: 98 (45.6%) with high-risk status and 117 (54.4%) with non-high-risk status (Table 1). Infection with hepatitis B virus was the most common etiology (55.3%), and 28.4% of patients had non-viral etiology. Of the patients with non-viral etiology, 40 patients had alcoholic cirrhosis and nineteen of them belonged to the high-risk group. There was no difference in the etiology between the high-risk and the non-high-risk group. A total of 186 patients (86.5%) were considered Child–Pugh class A, and 29 patients (13.5%) were considered Child–Pugh class B. The Barcelona Clinic Liver Cancer (BCLC) stage was B in 29 patients (13.5%) and C in 186 patients (86.5%). There were 129 patients (60%) with extrahepatic metastases in different locations (lung: 30; lymph node: 30; adrenal gland: 2; bone: 12; peritoneum: 7; and multiple sites: 48). Macrovascular invasion was present in 108 patients (50.2%), and the baseline serum AFP level exceeded 400 ng/mL in 90 patients (41.9%). The pre-treatment esophagogastroduodenoscopy results indicated that 108 patients (50.2%) had esophageal varices, and 15 of these patients (7.0%) were treated for variceal bleeding. A total of 140 patients (65.1%) received prior treatment for HCC (surgery, 13; RFA, 4; TACE, 67; combination treatment, 56). There were significant differences in baseline characteristics between the high-risk and non-high-risk populations. Most notably, the high-risk group had more patients of a younger age, advanced cancer (BCLC stage), and poor liver function based on Child–Pugh class (both *p* < 0.001). 

In total, 128 patients (59.5%) received neoadjuvant (*n* = 49, 38.3%) or concomitant (*n* = 79, 61.7%) radiation therapy. Most of the remaining patients refused to receive radiation therapy (*n* = 73), and other patients could not receive radiation therapy because they lacked an appropriate target site (*n* = 14). Baseline characteristics were not significantly different according to radiation therapy, except age, BCLC tumor stage, macrovascular invasion, prothrombin time, and neutrophil-to-lymphocyte ratio (NLR) in the total and non-high-risk populations (Tables S1 and S2). However, in the high-risk population, baseline characteristics were not significantly different between those who received and did not receive radiation therapy, except for macrovascular invasion (Table S3).

The target site in radiation therapy was a portal vein tumor thrombus [PVTT] (*n* = 78, 61.0%), a metastatic mass alone (*n* = 34, 26.6%), an intrahepatic mass alone (*n* = 9, 7.0%), an intrahepatic mass plus a metastatic mass (*n* = 4, 3.1%), or a PVTT plus a metastatic mass (*n* = 3, 2.3%). The median total radiation dose was 40 Gy (quartile 1 to quartile 3, 30–45 Gy). The target site in radiation therapy was significantly different between the high-risk and non-high-risk groups. PVTT was the most common target site in both groups (78.7% vs. 44.8% in high-risk vs. non-high-risk groups). Metastatic mass was also common as PVTT in the non-high-risk group (40.3%) but not in the high-risk group (9.8%).

### 3.2. Best Tumor Responses

Two patients did not undergo initial evaluations of the tumor response. Analysis of the best responses in the entire population indicated CR in 4 patients (1.9%), PR in 43 patients (20.0%), SD in 112 patients (52.1%), and PD in 56 patients (26%). The ORR was 21.9% and the DCR was 74.0% in the total population, the ORR was 23.5% and the DCR was 67.3% in the high-risk population, and the ORR was 20.5% and the DCR was 79.5% in the non-high-risk population (Table S4).

### 3.3. Disease Control Rate

Univariate analysis of the total population indicated that elevated AFP (>400 ng/mL, elevated DCP (>2000 mAU/mL), extrahepatic spread, liver infiltration of more than 50%, elevated NLR, and higher baseline ALBI grade were associated with worse DCR; multivariate analysis indicated that elevated AFP, extrahepatic spread, liver infiltration greater than 50%, and elevated NLR were associated with worse DCR (Table S5).

Univariate analysis of the high-risk population indicated that macrovascular invasion, Vp4 portal vein thrombus, liver infiltration > 50%, without radiation therapy, elevated NLR, and high ALBI grade were associated with worse DCR, and multivariate analysis indicated that liver infiltration > 50% was associated with worse DCR (Table S6). 

### 3.4. Progression-Free Survival

Analysis of the total population indicated that the median follow-up duration was 6.75 months (range 0.25–22). At the time of final data collection (June 30, 2022), the median number of chemotherapy cycles was seven (range 1–28) in the total population, five (range, 1–28) in patients who completed treatment (*n* = 134, 62.3%), and nine (range 1–28) in patients still receiving treatment (*n* = 81, 37.7%). At the end of the follow-up, patients who showed CR (*n* = 1), PR (*n* = 8), and SD (*n* = 35) at their best response evaluation showed progression.

**Figure 1:**
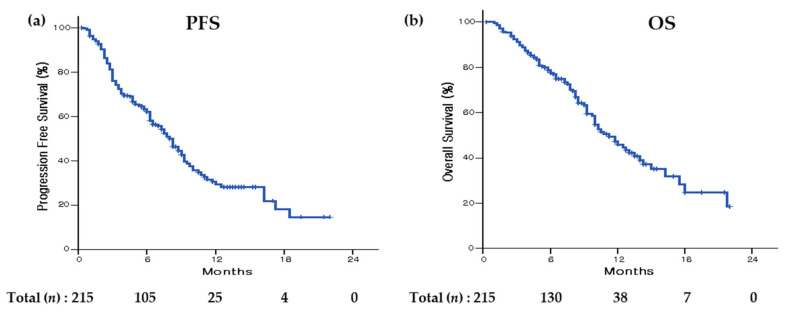
Overall survival and progression-free survival in the total population. In the total population, median PFS was 8.00 months (95% CI, 6.82–9.18) (**a**). During follow-up, 92 patients (42.8%) died, and median OS was 11.25 months (95% CI, 9.50–13.10) (**b**). PFS, progression-free survival; OS, overall survival.

**Figure 2:**
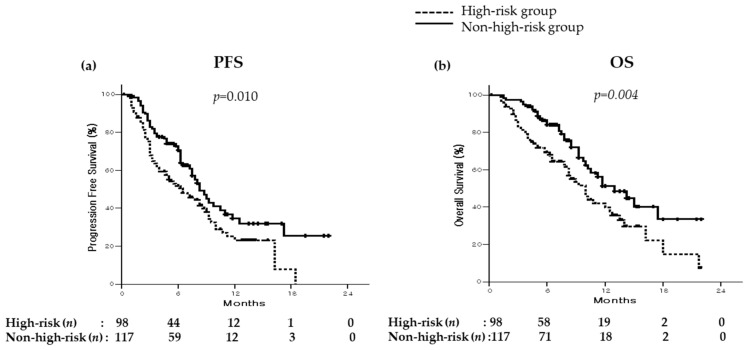
Overall survival and progression-free survival in the high-risk population and the non-high-risk population. (a) The high-risk population had a significantly shorter progression-free survival (PFS) than the non-high-risk population [median PFS of high-risk vs. non-high-risk population: 6.50 months (95% CI, 3.93–9.08) vs. 8.25 months (95% CI, 6.81–9.69); *p* = 0.010 (log-rank test)]. (**b**) The high-risk population had a significantly shorter OS than the non-high-risk population [median OS of high-risk vs. non-high-risk population: 10 months (95% CI, 8.19–11.82) vs. 13 months (95% CI, 9.44–16.56); *p* = 0.004 (log-rank test)].

Analysis of the total population indicated that median PFS was 8.00 months (95% CI, 6.82–9.18; Figure 1), and analysis of the high-risk and non-high-risk populations indicated the median PFS was 6.50 months (95% CI, 3.93–9.08) and 8.25 months (95% CI, 6.81–9.69), respectively. The high-risk population had a significantly shorter PFS than the non-high-risk population (*p* = 0.010; Figure 2).

Univariate analysis of the total population showed that Child–Pugh score, AFP level, DCP level, liver infiltration, varix, hemoglobin (Hb) level, NLR, and ALBI grade were associated with PFS, and multivariate analysis showed that elevated NLR and high ALBI grade were associated with shorter PFS (Table S7). 

Univariate analysis of the high-risk population showed that BCLC stage, DCP level, macrovascular invasion, liver infiltration, radiation therapy, Hb level, NLR, and ALBI grade were associated with PFS, while multivariate analysis showed that DCP level, radiation therapy, and ALBI grade were associated with PFS (Table S8). 

### 3.5. Overall Survival

During the follow-up, 92 patients (42.8%) died and median OS was 11.25 months (95% CI, 9.50–13.10) (Figure 1). Analysis of the high-risk population indicated that median OS was 10 months (95% CI, 8.19–11.82). The high-risk population had a significantly shorter OS than the non-high-risk population (median OS of non-high-risk population: 13 months [95% CI, 9.44–16.56); *p* = 0.004) (Figure 2).

Univariate analysis of the total population showed that Child–Pugh score, DCP level, Vp4 portal vein thrombosis, bile duct invasion, liver infiltration, prior local therapy, Hb level, aspartate aminotransferase (AST) level, presence of varices, NLR, and ALBI grade were associated with OS. Multivariate analysis showed that high DCP level, bile duct obstruction, low Hb level, elevated NLR, and high ALBI grade were associated with shorter OS (Table 2). 

Univariate analysis of the high-risk population showed that the Child–Pugh score, radiation therapy, Hb level, NLR, and ALBI grade were significantly associated with OS, and multivariate analysis showed that radiation therapy and lower ALBI grade were associated with longer OS (Table 3). 

### 3.6. Adverse Events

A total of 177 patients (82.3%) experienced AEs of any grade (Table 4 and Table S9). The most common AEs of any grade were proteinuria (23.7%), aminotransferase elevation (16.7%), thrombocytopenia (16.3%), neutropenia (10.7%), and hypertension (7.4%). Thirty-seven patients experienced at least one AE of grade 3 or higher (total, *n* = 64), and the most common AEs were variceal bleeding (*n* = 9), proteinuria (*n* = 4), hepatic encephalopathy (*n* = 4), and general weakness (*n* = 3). Twenty-five patients delayed ATE + BEV treatment at least once because of an AE, most commonly gastrointestinal (GI) bleeding (*n* = 8), general weakness (*n* = 4), and dermatitis (*n* = 3). GI bleeding occurred in 13 patients, gastric ulcer bleeding in 3 patients, esophageal variceal bleeding in 9 patients, and hemobilia in 1 patient. Hemoptysis occurred in two patients and hematuria occurred in two patients. Most cases of GI bleeding were controlled by endoscopic varix ligation or clipping.

A total of 13 patients discontinued ATE + BEV treatment permanently because of different AEs (pneumonitis, 2; limb weakness, 1; esophageal ulcer, 1; general weakness, 1; duodenal perforation, 1; autoimmune hepatitis, 1; hepatic encephalopathy, 3; aspartate aminotransferase elevation, 1; gastric ulcer bleeding and jaundice, 1; bowel necrosis, 1). Three patients died due to AEs, two from pneumonitis and one from duodenal perforation. The rates of total AEs and temporary or permanent drug discontinuation were similar in the high-risk and non-high-risk populations; however, there were significantly more grade 3 or higher AEs in the high-risk group (Table 4). Most cases of GI bleeding due to varices or ulcers occurred in the high-risk group (*n* = 12), except for one patient with ulcer bleeding (*p* < 0.001). The prevalence of GI bleeding (11/128 [8.5%] vs. 2/87 [2.3%]) and GI ulcers (7/128 [5.4%] vs. 1/87 [1.1%]) was higher in patients receiving radiation therapy than in those not receiving radiation therapy. Patients with Child–Pugh class B liver function had a lower prevalence of AEs (72.4%) and grade ≥ 3 AEs (10.3%).

### 3.7. Outcome after ATE + BEV Therapy

ATE + BEV treatment was discontinued in 134 patients (62.3%). The rate of treatment discontinuation was higher in the high-risk group than in the non-high-risk group (74.5% vs. 51.3%, *p* = 0.001). The specific causes of discontinuation were HCC progression (*n* = 83, 61.9%), impaired liver function (*n* = 26, 19.4%), AEs other than impaired liver function (*n* = 9, 6.7%), loss to follow-up (*n* = 6, 4.5%), patient refusal (*n* = 6, 4.5%), death due to non-HCC causes (*n* = 3, 2.2%), and liver resection after partial response (*n* = 1, 0.7%). Median OS after ATE + BEV discontinuation was 1.75 months (95% CI, 1.33–2.17), and it was significantly shorter in the high-risk group than in the non-high-risk group (1.50 months [95% CI, 0.97–2.03] vs. 3.00 months [95% CI, 1.58–4.42]; *p* = 0.021). Among the patients who permanently discontinued ATE + BEV, 46 (34.3%) received second-line therapy after progression (sorafenib, 44; regorafenib, 1; investigational agent, 1). One patient underwent liver resection because their HCC status improved with PR, as mentioned above. Most of the remaining patients received supportive care because of worsening liver function or performance status. Median OS after progression was significantly better in patients who received subsequent HCC treatment than in those who did not (5.75 months [95% CI, 3.50–8.00] vs. 1.00 month [95% CI, 0.54–1.46]; *p* < 0.001).

## 4. Discussion

This retrospective cohort study examined the real-world clinical efficacy and safety of ATE + BEV in patients with advanced HCC, especially in high-risk patients. Our total population consisted of 45.6% high-risk patients and 13.4% Child–Pugh class B patients. Baseline varices were present in 50.2% of patients. Therefore, a high percentage of our population were at high risk and had poor liver function.

We also examined the role of radiation therapy combined with ATE + BEV in the high-risk group. We found that radiation therapy did not affect tumor response or OS in our total population; however, multivariate analysis found that these therapies significantly improved PFS and OS in our high-risk population, and univariate analysis detected that the tumor response of the high-risk population significantly improved. This relatively high rate of success in the high-risk group might suggest the direct effectiveness of radiation therapy on PVTT (esp. Vp4), because the target site of radiation therapy in the high-risk group was mostly PVTT (80.3%), and several other reports have demonstrated the effectiveness of radiation therapy for Vp3 and Vp4 cases [19,20,21,22]. Some studies have shown the favorable efficacy and safety of the combination therapy of ATE + BEV and radiotherapy. Wang et al. analyzed 30 patients with PVTT that were treated with a combination of ATE + BEV and radiotherapy. They reported that median OS and PFS were 9.8 and 8.0 months, and the ORR was 76.6% [23]. Manzar et al. also analyzed the efficacy of 21 patients (of which 71.4% had PVTT) that were treated with a combination of ATE + BEV and radiotherapy; they reported that median OS was 16.1 months [24]. Both studies reported that the combination of ATE + BEV and radiotherapy showed tolerable and manageable toxicity. Jones-Pauely et al. suggested that combination of systemic and locoregional therapy might provide patients with large tumor burden opportunity from effective tumor control to downstaging [25]. However, this alone cannot explain all the success which occurred in the high-risk group because more than half of the high-risk group in this study had extrahepatic metastases that were not covered by radiation therapy. The authors suppose that tumor immunity is involved. Previous reports found that local extracorporeal radiation therapy enhanced the effect of immune checkpoint inhibitors when administered with different treatments in various non-clinical and clinical settings [26,27,28,29]. In these patients, local extracorporeal radiation therapy activates and promotes the maturation of antigen-presenting cells by altering various immune-related factors in cancer cells (ATP, GM-CSF, HMGB1, etc.), promoting their influx into the lymph nodes. This leads to the activation of T cells in the lymph glands, the influx of activated T cells in the vicinity of cancer cells (e.g., irradiated tumor and distant metastases), and killing of cancer cells [30,31,32] (Figure S1). Additionally, radiation is a method explored to make “cold tumors” hotter. Hotter tumors are more likely to be infiltrated by T cells, and such infiltrated CD8+ T cells could be activated by ICIs [33]. Li et al. showed that low-dose radiotherapy combined with ATE + BEV showed superior efficacy by mobilizing exhausted-like CD8+ T cells in preclinical HCC models [34]. Additional data would be needed to prove this hypothesis.

Our evaluation of the best responses indicated that the ORR and DCR were 21.9% and 74%, respectively. The median PFS and OS were 8.00 months and 11.25 months. These findings are comparable to the results of the IMbrave150 study, although our OS time was shorter [6]. Our results also showed that ATE + BEV was effective in terms of tumor response in high-risk patients. In fact, our high-risk group had a similar ORR and DCR as the non-high-risk group. These favorable ORR and DCR results might be due to the use of radiation therapy. Moreover, these favorable ORRs might be one reason for the better median OS (10 months) in the high-risk group compared with the results (7.6 months) in the updated data of the IMbrave150 study [15]. Tajiri et al. analyzed 18 studies (2937 patients) focused on ATE + BEV therapy in HCC patients and reported that ORR was the most significant contributor [35]. However, median OS in our study (11.25 months) was considerably shorter than reported in the updated analysis of the IMbrave150 study (19.2 months) [7] and in another real-world study (14.9 months) [36]. We believe this was because our total study population had a larger percentage of high-risk patients, and our high-risk group had a higher prevalence of poorer liver function and advanced BCLC stage and had much shorter OS than our non-high-risk group. Also, 8.6% of the non-high-risk group consisted of Child–Pugh class B patients. Notably, median PFS is comparable to previous studies [7,36], but OS after treatment discontinuation is shorter than that of another molecular target therapy [37]. Furthermore, only 34.3% of patients received second-line therapy after progression to ATE + BEV treatment, and the rest of the patients did not receive second-line therapy due to deterioration of liver function and performance status. The significantly shorter post-progression survival in these patients compared to those who received additional treatment may also have influenced the short survival time in this study. Lastly, follow-up duration of our study was much shorter than the one of IMbrave150 and from another real-world study (6.75 vs. 19.2 and 9 months, respectively) [7,36], which could partially explain the shorter OS in this study. Further OS data provided by a longer follow-up would be needed in future studies to refine those findings.

We found that the baseline NLR and ALBI grade were significant prognostic factors for PFS and OS. An elevated NLR indicates neutrophilia or lymphopenia, both of which are associated with pro-tumoral chronic inflammation or decreased antitumoral immune response [38]. Previous research reported associations of a high NLR and poor tumor response with worse outcomes in patients who received ATE + BEV combination treatment, although the exact NLR cut-off value differed among studies [39,40,41]. The baseline ALBI grade was associated with PFS and OS in both the overall and high-risk populations in this study. The ALBI grade has been considered more important than the Child–Pugh classification in HCC treatment because it is more objective and has been established based on a statistical method [42].

Anemia is not a well-known prognostic factor for HCC, and baseline Hb levels were significantly associated with OS in this study. Anemia is a common abnormality in patients with advanced cancer due to tumor progression or receipt of antitumor therapy [43] and is associated with poor prognosis in patients with various solid malignancies who receive chemotherapy, radiotherapy, or nivolumab monotherapy [44,45,46,47,48]. There could be several reasons why a low Hb level affects the efficacy of immunotherapy. For example, Zhao et al. reported that anemia could lead to a deficient T cell response and induce immunosuppression in patients with late-stage tumors [49]. In addition, hypoxia induced by a low Hb level can stimulate tumor growth and progression, decrease sensitivity to anticancer treatments, and lead to poor patient outcomes [50,51,52]. 

In this study, the rates of total AEs and temporary or permanent drug discontinuation were similar in the high-risk and non-high-risk populations as in the IMbrave150 subgroup analysis for the high-risk group [15]. In that study, the overall AE rate and a grade 3 or higher AE rate were similar in the high-risk ATE + BEV group, non-high-risk ATE + BEV group, and high-risk and non-high-risk sorafenib groups. However, grade 3 or higher hypertension was significantly more common in the ATE + BEV group than in the sorafenib group in both the high-risk and non-high-risk groups, and more grade 5 upper GI hemorrhage events were reported only in ATE + BEV group, especially the high-risk ATE + BEV group. In this study, there were significantly more grade 3 or higher AEs and GI bleeding in the high-risk group compared to the non-high-risk group. Therefore, when performing ATE + BEV combination treatment in HCC patients with high bleeding risk, such as varix bleeding, special attention to GI bleeding will be needed compared to sorafenib.

The present study has several limitations. First, the number of patients included in the study has been limited by the fact that ATE + BEV treatment was only partially reimbursed in the Republic of Korea during the period of the study. Second, this is a retrospective study, so some unintentional biases could exist. Some could come from the short follow-up period, some others from the fact that the radiation therapy was not performed as part of the randomized protocol, and finally some AEs may not have been reported. That being said, the rates of overall, grade 3 or higher AEs are comparable to those of previous real-world studies, suggesting our safety data are reliable. Despite these potential limitations, we acquired sufficient real-world data regarding median OS, median PFS, and tumor response in these patients.

## 5. Conclusions

ATE + BEV has become the first-line systemic treatment in HCC patients due to a remarkable improvement in survival outcomes compared to sorafenib on the basis of recent RCTs (IMbrave150) and several real-world data. However, there are still some unmet clinical needs, such as the poor efficacy of ATE + BEV in high-risk populations, such as patients with grade Vp4 portal vein thrombus, bile duct invasion, or more than 50% liver infiltration compared to non-high-risk populations (median OS, 7.6 vs. 22.8 months); however, in our study, ATE + BEV treatment provided consistent efficacy and tolerable safety in both groups. We analyzed the efficacy of radiotherapy combined with ATE + BEV in a high-risk population because some studies suggest the possibility of radiotherapy being able to enhance the therapeutic efficacy of ATE + BEV. In our study, we concluded ATE + BEV treatment had consistent efficacy and tolerability in both the total and high-risk population, unlike what was reported in the IMbrave150 study. Furthermore, radiation therapy combined with ATE + BEV might be helpful to improve PFS and OS in high-risk populations, and the safety of this combination is tolerable. Further prospective randomized studies are needed to confirm these results.

## Figures and Tables

**Table 1:** Baseline characteristics of the total population, high-risk population, and non-high-risk population.

Variables	Total (*n* = 215) *n* (%) or Median (Range)	High-Risk (*n* = 98) *n* (%) or Median (Range)	Non-High-Risk (*n* = 117) *n* (%) or Median (Range)	*p*-Value
Age in years	63 (39–92)	60.5 (39–92)	66 (43–86)	0.011
Male sex	182 (84.7)	84 (85.7)	98 (83.8)	0.692
Etiology				0.296
Hepatitis B	119 (55.3)	58 (59.2)	61 (52.1)	
Hepatitis C	33 (15.3)	10 (10.2)	23 (19.7)	
Hepatitis B + hepatitis C coinfection	2 (0.9)	1 (1.0)	1 (0.9)	
Non-viral	61 (28.4)	29 (29.6)	32 (27.4)	
ECOG performance status score				0.073
0	143 (66.5)	59 (60.2)	84 (71.8)	
1	72 (33.5)	39 (39.8)	33 (28.2)	
Child–Pugh classification				<0.001
A5	154 (71.6)	53 (54.1)	101 (86.3)	
A6	32 (14.9)	26 (26.5)	6 (5.1)	
B7	24 (11.2)	15 (15.3)	9 (7.7)	
B8	4 (1.9)	3 (3.1)	1 (0.9)	
B9	1 (0.5)	1 (1.0)	0 (0)	
Ascites	20 (9.3)	16 (16.3)	4 (3.4)	0.002
Barcelona Clinic Liver Cancer stage				<0.001
A	0	0	0	
B	29 (13.5)	4 (4.1)	25 (21.4)	
C	186 (86.5)	94 (95.9)	92 (78.6)	
Alpha-fetoprotein, ng/mL	190.8 (1.3–121,000)	610.1 (1.3–121,000)	81 (1.3–100,000)	<0.001
Alpha-fetoprotein > 400 ng/mL	90 (41.9)	52 (53.1)	38 (32.5)	0.002
DCP, mAU/ml	748 (13–615,936)	3722 (14.45–615,936)	184 (13–239,099)	<0.001
Macrovascular invasion	108 (50.2)	81 (82.7)	27 (23.1)	<0.001
Vp4 portal vein thrombus,	70 (32.6)	70 (71.4)	0 (0)	<0.001
Bile duct invasion	23 (10.7)	23 (23.5)	0 (0)	<0.001
Liver infiltration > 50%	48 (22.3)	48 (49.0)	0 (0)	<0.001
Extrahepatic metastasis	129 (60.0)	55 (56.1)	74 (63.2)	0.288
Prior local therapy for hepatocellular carcinoma	140 (65.1)	41 (41.8)	99 (84.6)	<0.001
Neoadjuvant or concomitant radiation therapy	128 (59.5)	61 (62.2)	67 (57.3)	0.459
Varices				
Present at baseline	108 (50.2)	59 (60.2)	49 (41.9)	0.007
Treated at baseline	15 (7.0)	12 (12.2)	3 (2.6)	0.006
WBC (/mm^3^)	5760 (1390–27,540)	6265 (1690–27,540)	5300 (1390–25,440)	0.009
Hb (g/dL)	12.7 (6.5–20.5)	12.4 (6.9–17.6)	13 (6.5–20.5)	0.023
AST (U/L)	50 (16–550)	65.5 (16–550)	37 (16–331)	<0.001
ALT (U/L)	29 (6–349)	35 (9–281)	26 (6–349)	0.002
Albumin (g/dL)	3.9 (2.1–5.0)	3.7 (2.5–4.6)	4.1 (2.1–5.0)	<0.001
Total bilirubin (mg/dL)	0.9 (0.25–6.60)	1.0 (0.3–6.6)	0.8 (0.3–2.5)	0.002
Prothrombin time (INR)	1.12 (0.88–1.74)	1.14 (0.93–1.74)	1.10 (0.88–1.64)	<0.001
Neutrophil-to-lymphocyte ratio	3.06 (0.25–19.73)	3.73 (1.38–19.73)	2.72 (0.25–12.96)	0.036
≤2.25	64 (29.9)	22 (22.7)	42 (35.9)	
>2.25	150 (70.1)	75 (77.3)	75 (64.1)	
ALBI grade				<0.001
1	99 (46)	27 (27.6)	72 (61.5)	
2	109 (50.7)	67 (68.4)	42 (35.9)	
3	7 (3.3)	4 (4.1)	3 (2.6)	

Abbreviations: ECOG, Eastern Cooperative Oncology Group; DCP, des-gamma-carboxy prothrombin; WBC, white blood cells; Hb, hemoglobin; AST, aspartate aminotransferase; ALT, alanine aminotransferase; ALBI, albumin–bilirubin.

**Table 2:** Univariate and multivariate analysis of factors associated with overall survival in the total population.

Subgroup	Events/Patients	Median OS, Months (95% CI)	*p*-Value *	*p*-Value **
All patients	92/215	11.25 (9.50–13.10)		
Age, years < 65	48/113	11.25(8.18–14.32)	0.777	
≥65	44/102	11.75 (9.69–13.81)		
Sex			0.815	
Male	74/182	11.25 (9.25–13.25)		
Female	18/33	13 (8.41–17.59)		
Etiology			0.617	
Hepatitis B	54/119	10.75 (8.13–13.37)		
Hepatitis C	9/33	14.25 (9.23–19.27)		
Hepatitis B + hepatitis C	0/2	-		
Non-viral	29/61	9.75 (8.55–10.95)		
ECOG performance status			0.169	
0	58/143	12.75 (9.94–15.56)		
1	34/72	10 (7.82–12.18)		
Child–Pugh score			<0.001	0.408
5A	50/154	14.00 (10.41–17.59)		
6A	21/32	6.50 (5.14–7.86)		
7B	17/24	8.00 (2.07–13.93)		
8B	4/4	5.75 (0–13.10)		
9B	0/1	-		
Ascites			0.001	0.079
Absent at baseline	78/195	11.75 (9.13–14.37)		
Present at baseline	14/20	7.25 (2.96–11.54)		
Barcelona Clinic Liver Cancer stage			0.204	
B	6/29	14.25 (-)		
C	86/186	10.50 (8.83–12.17)		
Alpha-fetoprotein at baseline			0.172	
≤400 ng/mL	46/125	12.00 (9.32–14.68)		
>400 ng/ml	46/90	10.00 (8.02–11.98)		
DCP at baseline (mAU/mL)			0.023	0.034
≤2000	43/123	12.75 (10.31–15.19)		
>2000	47/88	8.50 (6.69–10.31)		
Macrovascular invasion at baseline			0.085	
No	37/107	13.00 (9.87–16.19)		
Yes	55/108	10.00 (8.78–11.22)		
Extrahepatic metastasis			0.500	
No	33/86	11.75 (9.16–14.34)		
Yes	59/129	10.75 (7.37–14.13)		
Vp4 portal vein thrombus			0.001	0.710
No	52/145	14.00 (10.90–17.10)		
Yes	40/70	9.25 (7.76–10.74)		
Bile duct invasion			0.014	0.034
No	76/192	11.75 (9.49–14.01)		
Yes	16/23	8.25 (3.44–13.06)		
Liver infiltration > 50%			0.002	0.923
No	61/167	12.00 (9.74–14.26)		
Yes	31/48	8.25 (5.39–11.11)		
Prior local therapy			0.007	0.751
No	38/75	9.75 (7.60–11.90)		
Yes	54/140	12.75 (9.47–15.53)		
Neoadjuvant or concomitant radiation therapy			0.305	
No	35/87	10.50 (7.60–13.40)		
Yes	57/128	12.00 (8.76–15.24)		
Hb (g/dL)			0.001	0.013
≤12.5	57/99	9.25 (7.65–10.85)		
>12.5	35/116	14.00 (9.57–18.43)		
AST (U/L)			0.001	0.490
≤40	26/81	15.00 (8.73–21.27)		
>40	66/134	10.00 (8.38–11.62)		
Varices			0.004	0.504
Absent at baseline	35/107	16.25 (10.92–21.58)		
Present at baseline	60/108	10.00 (9.02–10.98)		
Neutrophil-to-lymphocyte ratio			0.001	0.017
≤2.25	18/64	18.00 (10.38–25.62)		
>2.25	74/150	10.00 (8.64–11.36)		
ALBI grade			<0.001	<0.001
1	23/99	17.50 (13.34–21.66)		
2	62/109	8.25 (7.48–9.02)		
3	7/7	5.00 (0.00–13.34)		

Abbreviations: CI, confidence interval; ECOG, Eastern Cooperative Oncology Group; DCP, des-gamma-carboxy prothrombin; Hb, hemoglobin; AST, aspartate aminotransferase; ALBI, albumin–bilirubin. * *p* value by univariate analysis; ** *p* value by multivariate analysis.

**Table 3:** Univariate and multivariate analysis of factors associated with overall survival in the high-risk population.

Subgroup	Events/Patients	Median OS, Months (95% CI)	*p*-Value *	*p*-Value **
All patients	54/98	10 (8.19–11.82)		
Age, years < 65	30/60	10 (5.27–14.73)	0.326	
≥65	24/38	10 (7.00–13.00)		
Sex			0.416	
Male	45/84	9.25 (7.78–10.72)		
Female	9/14	10.25 (5.68–14.82)		
Etiology			0.161	
Hepatitis B	33/58	10.25 (6.21–14.29)		
Hepatitis C	3/10	21.75 (-)		
Hepatitis B + hepatitis C	0/1	-		
Non-viral	18/29	8.25 (5.13–11.37)		
ECOG performance status			0.187	
0	31/59	10.00 (7.84–12.16)		
1	23/39	8.50 (4.07–12.93)		
Child–Pugh score			0.012	0.516
5A	23/53	13.50 (9.08–17.92)		
6A	17/26	6.50 (4.74–8.26)		
7B	11/15	5.75 (1.32–10.18)		
8B	3/3	9.00 (3.80–14.20)		
9B	0/1	-		
Ascites			0.239	
Absent at baseline	43/82	10.00 (7.88–12.12)		
Present at baseline	11/16	7.75 (0.70–14.80)		
Barcelona Clinic Liver Cancer stage			0.316	
B	3/4	4.00 (1.80–6.20)		
C	51/94	10.00 (8.23–11.77)		
Alpha-fetoprotein at baseline			0.833	
≤400 ng/mL	26/46	10.00 (6.73–13.27)		
>400 ng/ml	28/52	8.50 (6.37–10.63)		
DCP at baseline (mAU/mL)			0.282	
≤2000	19/39	12.00 (8.90–15.10)		
>2000	33/56	8.25 (6.30–10.20)		
Macrovascular invasion at baseline			0.104	
No	11/17	4.25 (0.08–8.42)		
Yes	43/81	10.00 (8.03–11.97)		
Extrahepatic metastasis			0.801	
No	25/43	9.00 (7.08–10.92)		
Yes	29/55	10.00 (5.13–14.87)		
Vp4 portal vein thrombus			0.328	
No	14/28	14.00 (6.45–21.55)		
Yes	40/70	9.25 (7.76–10.74)		
Bile duct invasion			0.165	
No	38/75	10.00 (7.83–12.17)		
Yes	16/23	8.25 (3.44–13.06)		
Liver infiltration over 50%			0.127	
No	23/50	10.25 (6.52–13.98)		
Yes	31/48	8.25 (5.39–11.11)		
Prior local therapy			0.272	
No	30/57	10.00 (5.88–14.12)		
Yes	24/41	10.00 (6.63–13.37)		
Neoadjuvant or concomitant radiation therapy			0.001	0.001
No	24/37	5.75 (3.24–8.26)		
Yes	30/61	12.00 (9.33–14.67)		
Varices			0.514	
Absent at baseline	17/39	16.25 (-)		
Present at baseline	37/59	10.00 (8.34–11.66)		
Hb (g/dL)			0.016	0.057
≤12.5	34/50	8.00 (6.12–9.88)		
>12.5	20/48	14.00 (10.37–17.63)		
AST (U/L)			0.752	
≤40	9/14	9.25 (6.88–11.62)		
>40	45/84	10.00 (7.38–12.62)		
Varix			0.514	
Absent	17/39	16.25 (-)		
Present	37/59	10.00 (8.34–11.66)		
Neutrophil-to-lymphocyte ratio			0.009	0.163
≤2.25	7/22	18.00 (9.77–26.23)		
>2.25	47/75	8.25 (5.69–10.81)		
ALBI grade			<0.001	0.007
1	9/27	16.25 (12.00–20.50)		
2	41/67	8.25 (6.18–10.32)		
3	4/4	1.75 (0–5.91)		

Abbreviations: CI, confidence interval; ECOG, Eastern Cooperative Oncology Group; DCP, des-gamma-carboxy prothrombin; Hb, hemoglobin; AST, aspartate aminotransferase; ALBI, albumin–bilirubin. * *p* value by univariate analysis; ** *p* value by multivariate analysis.

**Table 4:** Adverse events in the total population, high-risk population, and non-high-risk population.

Adverse Event	Total (*n* = 215), *n* (%)	High-Risk (*n* = 98), *n* (%)	Non-High-Risk (*n* = 117), *n* (%)	*p*-Value
Any adverse event	177 (82.3)	79 (80.6)	98 (83.8)	0.593
Grade 3 or 4 adverse event	37 (17.2)	24 (24.5)	13 (11.1)	0.019
Gastrointestinal bleeding	13 (6.04)	12 (12.24)	1 (0.85)	<0.001
Temporary discontinuation	25 (11.6)	13 (13.2)	12 (10.2)	0.519
Permanent discontinuation	13 (6.0)	7 (7.1)	6 (5.1)	0.549

## Data Availability

The datasets used and/or analyzed during the current study are available from the corresponding author on reasonable request.

## Supplementary Materials

The following supporting information can be downloaded at: https://www.mdpi.com/article/10.3390/cancers16040838/s1, Table S1: Comparison of baseline characteristics between patients with radiation and without radiation therapy in the total population; Table S2: Comparison of baseline characteristics between patients with radiation and without radiation therapy in the non-high-risk population; Table S3: Comparison of baseline characteristics between patients with radiation and without radiation therapy in the high-risk population; Table S4: Treatment responses of the total population, high-risk population, and non-high-risk population based on RECIST version 1.1; Table S5: Univariate and multivariate analysis of factors associated with disease control rate in the total FacciabeneFacciabenepopulation; Table S6: Univariate and multivariate analysis of factors associated with disease control rate in the high-risk population; Table S7: Univariate and multivariate analysis of factors associated with progression-free survival (PFS) in the total population; Table S8: Univariate and multivariate analysis of factors associated with progression-free survival (PFS) in the high-risk population; Table S9: Profiles of adverse events in the total population; Figure S1: The mechanism of atezolizumab plus bevacizumab and radiation therapy combined with atezolizumab plus bevacizumab against hepatocellular carcinoma; Figure S2: Comprehensive flow sheet; Figure S3: Infographic abstract; Figure S4: Comparative finding of pre and post treatment. [53,54,55,56,57,58,59,60,61,62,63,64].
