# Supplementary material for: Outcome of Atezolizumab Plus Bevacizumab Combination Therapy in High-Risk Patients with Advanced Hepatocellular Carcinoma
Source: Cancers (Basel). 2024 Feb 19;16(4):838. doi: 10.3390/cancers16040838 (PMC10887033; doi:10.3390/cancers16040838)
Supplement: Supplementary file 1 [file cancers-16-00838-s001.zip › cancers-2842091-supplementary.pdf]

Supplementary Materials

Outcome of Atezolizumab plus Bevacizumab Combination Therapy in High-Risk Patients with Advanced Hepatocellular Carcinoma

Sang Youn Hwang [1,†], Hyun Young Woo [2,†], Jeong Heo [2,*,‡], Hyung Jun Kim [1], Young Joo Park [2], Ki Youn Yi [2], Yu Rim Lee [3], Soo Young Park [3], Woo Jin Chung [4], Byoung Kuk Jang [4] and Won Young Tak [3,*,‡]

Table S1……………………………………………………………………..2

Table S2…………………………… ………………………..………………….5

Table S3…………………………..…………………………………………….8

Table S4…………………………..……………………………………………11

Table S5…………………………..……………………………………………12

Table S6…………………………………………..……………………………15

Table S7…………………………………………………….…………………18

Table S8…………………………………………..……………………………22

Table S9…………………………...……………………………………………26

Figure S1………………………………………………………………………..29

Figure S2………………………………………………………………………..30

Figure S3………………………………………………………………………..31

Figure S4………………………………………………………………………..32

References………………………………………………………………………..33

Table S1. Comparison of baseline characteristics between patients with radiation and without radiation therapy in the total population.

Variables	Radiation (n=128) n (%) or median (range)	Without Radiation (n=87) n (%) or median (range)	p-Value
Age in years	61.5 (40-84)	66 (39-93)	0.006
Male sex	108 (84.4)	74 (85.1)	1.000
Etiology			0.111
Hepatitis B	76 (59.4)	43 (49.4)	
Hepatitis C	21 (16.4)	12 (13.8)	
Hepatitis B + hepatitis C coinfection	0 (0)	2 (2.3)	
Non-viral	31 (24.2)	30 (34.5)	
ECOG performance status score			0.055
0	92 (71.9)	51 (58.6)	
1	36 (28.1)	36 (41.4)	
Child-Pugh classification			0.594
A5	94 (73.4)	60 (69)	
A6	20 (15.6)	12 (13.8)	
B7	12 (9.4)	12 (13.8)	

B8	2 (1.6)	2 (2.3)	
B9	0 (0)	1 (1.1)	
Barcelona Clinic liver cancer stage			<0.001
A	0	0	
B	7 (5.5)	22 (25.3)	
C	121 (94.5)	65 (74.7)	
Alpha-fetoprotein, ng/mL	610.1 (1.3-121,000)	81 (1.3-100,000)	0.712
Alpha-fetoprotein>400 ng/mL	55 (43)	35 (40.2)	0.778
DCP, mAU/ml	3722 (14.45-615,936)	184 (13-239,099)	0.626
Macrovascular invasion	77 (60.2)	31 (35.6)	0.001
Vp4 portal vein thrombus,	45 (35.2)	25 (28.7)	0.375
Bile duct invasion	16 (12.5)	7 (8.0)	0.372
Liver infiltration>50%	26 (20.3)	22 (25.3)	0.408
High risk group	61 (47.7)	37 (42.5)	0.488
Extrahepatic metastasis	81 (63.3)	48 (55.2)	0.258
Prior local therapy for Hepatocellular carcinoma	85 (66.4)	55 (63.2)	0.663
Varices			
Present at baseline	67 (52.3)	41 (47.1)	0.489

Treated at baseline	9 (7.0)	6 (6.9)	1.000
WBC (/mm3)	5,490 (1,390-15,500)	5,930 (2,510-27,540)	0.149
Hb (g/dL)	12.8 (6.5-20.5)	12.6 (8.1-17.6)	0.524
AST (U/L)	51 (16-550)	50 (16-373)	0.927
ALT (U/L)	29.5 (8-349)	26 (6-125)	0.244
Albumin (g/dL)	3.9 (2.6-4.8)	3.9 (2.1-5.0)	0.482
Total bilirubin (mg/dL)	0.9 (0.2-6.6)	0.9 (0.3-4.0)	0.695
Prothrombin time (INR)	1.10 (0.88-1.56)	1.14 (0.92-1.74)	0.022
Neutrophil to lymphocyte ratio			0.089
≤2.25	28 (22)	36 (41.4)	0.004
>2.25	99 (78)	51 (58.6)	
ALBI grade			0.228
1	59 (46.1)	40 (46)	
2	67 (52.3)	42 (48.3)	
3	2 (1.6)	5 (5.7)	

Abbreviations: ECOG, Eastern Cooperative Oncology Group; DCP, des-gamma-carboxy prothrombin; WBC, white blood cells; Hb, hemoglobin; ANC, absolute neutrophil count; AST, aspartate aminotransferase; ALT, alanine aminotransferase; ALBI, albumin-bilirubin.

Table S2. Comparison of baseline characteristics between patients with radiation and without radiation therapy in the non-high-risk population.

Variables	Radiation (*n*=67)<br>*n* (%) or median (range)	Without Radiation (*n*=50)<br>*n* (%) or median (range)	*p*-Value
Age in years	65 (47-81)	68.5 (43-86)	0.044
Male sex	56 (83.6)	42 (84.0)	
Etiology			0.320
Hepatitis B	38 (56.7)	23 (46.0)	
Hepatitis C	14 (20.9)	9 (18.0)	
Hepatitis B + hepatitis C coinfection	0 (0)	1 (2.0)	
Non-viral	15 (22.4)	17 (34.0)	
ECOG performance status score			0.299
0	51 (76.1)	33 (66)	
1	16 (23.9)	17 (34)	
Child-Pugh classification			
A5	59 (88.1)	42 (84.0)	
A6	3 (4.5)	3 (6.0)	
B7	5 (7.5)	4 (8.0)	

B8	0 (0)	1 (2.0)	
Barcelona Clinic liver cancer stage			<0.001
A	0	0	
B	6 (9.0)	19 (38.0)	
C	61 (91.0)	31 (62.0)	
Alpha-fetoprotein, ng/mL	81.3 (1.3-100,000)	74.7 (1.3-46,405)	0.572
Alpha-fetoprotein>400 ng/mL	23 (34.3)	15 (30.3)	0.692
DCP, mAU/ml	279 (13-68,999)	143.5 (14.0-239,099)	0.457
Macrovascular invasion	22 (32.8)	5 (10.0)	0.004
Extrahepatic metastasis	45 (67.2)	29 (58.0)	0.337
Prior local therapy for Hepatocellular carcinoma	58 (86.6)	41 (82.0)	0.606
Varices			
Present at baseline	29 (43.3)	20 (40.0)	0.850
Treated at baseline	1 (1.5)	2 (4.0)	0.575
WBC (/mm3)	5,090 (1,390-11,150)	5,675 (2,510-24,440)	0.587
Hb (g/dL)	12.9 (6.5-20.5)	13.0 (8.3-17.0)	0.862
AST (U/L)	38 (18-331)	35 (16-156)	0.274
ALT (U/L)	28 (8-349)	24.5 (6-91)	0.190
Albumin (g/dL)	4.1 (2.7-4.7)	4.0 (2.1-5.0)	0.636

Total bilirubin (mg/dL)	0.80 (0.25-2.49)	0.80 (0.30-2.40)	0.842
Prothrombin time (INR)	1.08 (0.88-1.44)	1.13 (0.92-1.64)	0.074
Neutrophil to lymphocyte ratio	3.22 (0.25-11.85)	2.15 (0.77-12.96)	<0.001
≤2.25	13 (19.4)	29 (58.0)	<0.001
>2.25	54 (80.6)	21 (42.0)	
ALBI grade			0.673
1	41 (61.2)	31 (62.0)	
2	25 (37.3)	17 (34.0)	
3	1 (1.5)	2 (4.0)	

Abbreviations: ECOG, Eastern Cooperative Oncology Group; DCP, des-gamma-carboxy prothrombin; WBC, white blood cells; Hb, hemoglobin; ANC, absolute neutrophil count; AST, aspartate aminotransferase; ALT, alanine aminotransferase; ALBI, albumin-bilirubin.

Table S3. Comparison of baseline characteristics between patients with radiation and without radiation therapy in the high-risk population.

Variables	Radiation (*n*=61) *n* (%) or median (range)	Without Radiation (*n*=37) *n* (%) or median (range)	*p*-Value
Age in years	58 (40-84)	65 (39-92)	0.054
Male sex	52 (85.2)	32 (86.5)	1.000
Etiology			0.426
Hepatitis B	38 (62.3)	20 (54.1)	
Hepatitis C	7 (11.5)	3 (8.1)	
Hepatitis B + hepatitis C coinfection	0 (0)	1 (2.7)	
Non-viral	16 (26.2)	13 (35.1)	
ECOG performance status score			0.089
0	41 (67.2)	18 (48.6)	
1	20 (32.8)	19 (51.4)	
Child-Pugh classification			0.455
A5	35 (57.4)	18 (48.6)	
A6	17 (27.9)	9 (24.3)	
B7	7 (11.5)	8 (21.6)	

B8	2 (3.3)	1 (2.7)	
B9	0 (0)	1 (2.7)	
Barcelona Clinic liver cancer stage			0.149
A	0	0	
B	1 (1.6)	3 (8.1)	
C	60 (98.4)	34 (91.9)	
Alpha-fetoprotein, ng/mL	670.0 (1.3-102,687)	550.2 (2.2-121,000)	0.673
Alpha-fetoprotein≥400 ng/mL	32 (52.5)	20 (54.1)	
DCP, mAU/ml	2531.5 (16.0-615,936)	14648.5 (14.4-406,124)	0.004
Macrovascular invasion	55 (90.2)	26 (70.3)	0.025
Vp4 portal vein thrombus,	45 (73.8)	25 (67.6)	0.645
Bile duct invasion	16 (26.2)	7 (18.9)	0.469
Liver infiltration>50%	26 (42.6)	22 (59.5)	0.145
Extrahepatic metastasis	36 (59.0)	19 (51.4)	0.531
Prior local therapy for Hepatocellular carcinoma	27 (44.3)	14 (37.8)	0.673
Varices			
Present at baseline	38 (62.3)	21 (56.8)	0.672
Treated at baseline	8 (13.1)	4 (10.8)	1.000

WBC (/mm3)	6,060 (2,940-15,500)	6,670 (2,940-27,540)	0.059
Hb (g/dL)	12.6 (6.9-17.5)	11.7 (8.1-17.6)	0.326
AST (U/L)	63 (16-550)	76 (31-373)	0.147
ALT (U/L)	36 (9-281)	33 (10-125)	0.968
Albumin (g/dL)	3.7 (2.6-4.6)	3.6 (2.5-4.6)	0.313
Total bilirubin (mg/dL)	0.91 (0.27-6.60)	1.00 (0.41-4.00)	0.301
Prothrombin time (INR)	1.14 (0.93-1.56)	1.14 (0.93-1.74)	0.095
Neutrophil to lymphocyte ratio	3.64 (1.38-15.09)	4.01 (1.42-19.73)	0.109
≤2.25	15 (25.0)	7 (18.9)	0.620
>2.25	45 (75.0)	30 (81.0)	
ALBI grade			0.274
1	18 (29.5)	9 (24.3)	
2	42 (68.9)	25 (67.6)	
3	1 (1.6)	3 (8.1)	

Abbreviations: ECOG, Eastern Cooperative Oncology Group; DCP, des-gamma-carboxy prothrombin; WBC, white blood cells; Hb, hemoglobin; ANC, absolute neutrophil count; AST, aspartate aminotransferase; ALT, alanine aminotransferase; ALBI, albumin-bilirubin.

Table S4. Treatment responses of the total population, high-risk population, and non-high-risk population based on RECIST version 1.1.

Best Responses	Rate		
	Overall population (*n*=215)	High risk (*n*=98)	Non-high risk (*n*=117)
Complete response	4 (1.9)	0 (0)	4 (3.4)
Partial response	43 (20.0)	23 (23.5)	20 (17.1)
Stable disease	112 (52.1)	43 (43.9)	69 (59.0)
Progressive disease	56 (26.0)	32 (32.6)	24 (20.5)
Objective response rate	47 (21.9)	23 (23.5)	24 (20.5)
Disease control rate	159 (74.0)	66 (67.3)	93 (79.5)

Abbreviations: RECIST, Response Evaluation Criteria in Solid Tumors.

Table S5. Univariate and multivariate analysis of factors associated with disease control rate in the total population.

Subgroup	Responders/n	DCR (%)	*p*-Value *	*p*-Value **
All patients	159/215	73.9		
Age, years <65	78/113	69	0.126	
≥65	81/102	79.4		
Sex			0.252	
Male	137/182	75.2		
Female	22/33	66.7		
Etiology			0.365	
Hepatitis B	84/119	70.5		
Hepatitis C	27/33	81.8		
Hepatitis B + hepatitis C	1/2	50		
Non-viral	47/61	77		
ECOG performance sta-			0.677	
tus	104/143	76.5		
0	55/72	69.6		
1				
Child-Pugh class			0.375	
A	140/186	75.7		
B	19/29	65.5		
Child-Pugh score			0.206	
5A	119/154	77.2		
6A	21/32	65.6		
7B	15/24	62.5		
8B	4/4	100		
9B	0/1	0		
Ascites			0.781	

Absent at baseline	146/195	74.9		
Present at baseline	13/20	65		
Barcelona Clinic Liver Cancer stage			0.124	
B	25/29	86.2		
C	134/186	72.8		
Alpha-fetoprotein at baseline (ng/ml)			0.001	0.029
≤400	103/125	82.4		
>400	56/90	62.2		
DCP at baseline			0.031	0.933
(mAU/ml)	97/123	78.8		
≤2000	59/88	67.0		
>2000				
Macrovascular invasion at baseline			0.968	
No	80/107	74.8		
Yes	79/108	73.1		
Extrahepatic metastasis			0.015	0.036
No	71/86	82.5		
Yes	88/129	68.2		
Vp4 portal vein thrombus			0.449	
No	106/145	73.1		
Yes	53/70	75.7		
Bile duct invasion			0.108	
No	145/192	75.5		
Yes	14/23	60.9		
Liver infiltration over			<0.001	0.005

50%	135/167	80.7		
No	24/48	51.0		
Yes				
Prior local therapy			0.620	
No	53/74	71.6		
Yes	106/141	75.1		
Neoadjuvant or concomitant radiation therapy			0.481	
No				
Yes	62/87	71.2		
	97/128	75.7		
Neutrophil to lymphocyte ratio			0.002	0.035
≤2.25	57/64	89.1		
>2.25	101/150	67.3		
ALBI grade			0.020	0.273
1	81/99	81.8		
2	74/109	69.2		
3	4/7	57.1		

Abbreviations: DCR, disease control rate; ECOG, Eastern Cooperative Oncology Group; DCP, des-gamma-carboxy prothrombin; ALBI, albumin-bilirubin.

**p* value by univariate analysis

** *p* value by multivariate analysis

Table S6. Univariate and multivariate analysis of factors associated with disease control rate in the high-risk population.

Subgroup	Responders/*n*	DCR (%)	*p*-Value *	*p*-Value **
All patients	66/98	67.3		
Age, years <65	37/60	61.6	0.195	
≥65	29/38	76.3		
Sex			0.758	
Male	57/84	67.8		
Female	9/14	64.3		
Etiology			0.389	
Hepatitis B	38/58	65.5		
Hepatitis C	6/10	60		
Hepatitis B + Hepatitis C	0/1	0		
Non-viral	22/29	75.9		
ECOG performance status			0.308	
0	36/49	73.5		
1	30/49	61.2		
Child-Pugh class			0.438	
A	55/79	69.6		
B	11/19	57.8		
Child-Pugh score			0.356	
5A	38/53	71.7		
6A	17/26	65.3		
7B	8/15	53.3		
8B	3/3	100		
9B	0/1	0		
Ascites			1.000	

Absent at baseline	56/82	68.3		
Present at baseline	10/16	62.5		
Barcelona Clinic Liver Cancer stage			0.054	
B	1/4	25.0		
C	65/94	69.1		
Alpha-fetoprotein at baseline (ng/ml)			0.177	
≤400	34/46	73.9		
>400	32/52	61.5		
DCP at baseline (mAU/ml)			0.246	
≤2000	28/39	71.7		
>2000	36/56	64.2		
Macrovascular invasion at baseline			0.001	0.692
No	6/17	35.3		
Yes	60/81	74.1		
Extrahepatic metastasis			0.165	
No	32/43	74.4		
Yes	34/55	61.8		
Vp4 portal vein thrombus			0.002	0.311
No	13/28	46.4		
Yes	53/70	75.7		
Bile duct invasion			0.350	
No	52/75	69.3		
Yes	14/23	60.9		
Liver infiltration over			<0.001	0.007

50%				
No	42/50	84.0		
Yes	24/48	50.0		
Prior local therapy			0.503	
No	40/57	70.1		
Yes	26/41	63.4		
Neoadjuvant or concomitant radiation therapy			0.031	0.315
No				
Yes	20/37	54.1		
	46/61	75.4		
Neutrophil to lymphocyte ratio			0.019	0.148
≤2.25	20/22	90.9		
>2.25	45/75	60.0		
ALBI grade			0.033	0.260
1	23/27	85.2		
2	41/67	61.2		
3	2/4	50		

Abbreviations: DCR, disease control rate; ECOG, Eastern Cooperative Oncology Group; DCP, des-gamma-carboxy prothrombin; ALBI, albumin-bilirubin.

**p* value by univariate analysis

** *p* value by multivariate analysis

Table S7. Univariate and multivariate analysis of factors associated with progression-free survival (PFS) in the total population.

Subgroup	Events/ patients	Median PFS, months (95% CI)	*p*-Value *	*p*-Value **
All patients	122/215	8.00 (6.82-9.18)		
Age, years <65	71/113	7.5 (6.06-8.94)	0.125	
≥65	51/102	35 (5.32-12.18)		
Sex			0.059	
Male	96/182	8.75 (7.47-10.03)		
Female	26/33	6.25 (3.14-9.36)		
Etiology			0.336	
Hepatitis B	74/119	7 (5.35-8.65)		
Hepatitis C	14/33	10.5 (7.00-14.00)		
Hepatitis B + hepatitis C	1/2	1.25 (-)		
Non-viral	33/61	8.25 (5.63-10.87)		
ECOG performance status			0.511	
0	79/143	8.25 (6.86-9.64)		
1	43/72	7.25 (5.49-9.01)		
Child-Pugh score			0.001	0.828
5A	76/154	35 (31.05-38.95)		
6A	23/32	19 (9.95-28.05)		
7B	20/24	13 (4.6-21.40)		
8B	3/4	36 (15.20-56.80)		
9B	0/1	-		
Ascites			0.020	0.233
Absent at baseline	107/195	8.25 (7.14-9.36)		
Present at baseline	15/20	4.00 (1.77-6.23)		

Barcelona Clinic Liver Cancer stage			0.084	
B	9/29	11.75 (6.72-16.78)		
C	113/186	7.5 (6.24-8.76)		
Alpha-fetoprotein at baseline (ng/ml)			0.008	0.184
≤400 ng/ml	61/125	9 (8.00-10.00)		
>400 ng/ml	61/90	5.25 (2.94-7.56)		
DCP at baseline (mAU/ml)			0.004	0.119
≤2000	58/123	9.25 (8.24-10.26)		
>2000	61/88	5.75 (3.89-7.61)		
Macrovascular invasion at baseline			0.074	
No	51/107	9 (6.44-11.56)		
Yes	71/108	7.5 (6.11-8.89)		
Extrahepatic metastasis			0.099	
No	43/86	9.25 (8.28-10.22)		
Yes	79/129	6.5 (5.26-7.74)		
Vp4 portal vein thrombus			0.165	
No	76/145	8.25 (6.74-9.76)		
Yes	46/70	7.75 (5.71-9.79)		
Bile duct invasion			0.099	
No	104/192	8.25 (7.02-9.48)		
Yes	18/23	4.75 (2.01-7.49)		
Liver infiltration over 50%			<0.001	0.091
No	83/167	8.75 (7.93-9.57)		

Yes	39/48	3.00 (2.36-3.89)		
Prior local therapy			0.076	
No	47/75	6.25 (4.28-8.22)		
Yes	75/140	8.74 (7.88-9.62)		
Neoadjuvant or concomitant radiation therapy			0.495	
No				
Yes	46/87	7 (5.25-8.75)		
	76/128	8.25 (7.10-9.40)		
Varices			0.033	0.934
absent at baseline	50/107	9.26 (6.68-11.82)		
present at baseline	72/108	7.25 (5.98-8.52)		
Hb level			0.003	0.265
≤12.5	68/99	6.25 (4.86-7.64)		
>12.5	54/116	9 (7.60-10.40)		
Neutrophil to lymphocyte ratio			0.001	0.004
≤2.25	26/64	11.25 (7.90-14.60)		
>2.25	96/150	6.50 (5.14-7.86)		
ALBI grade			<0.001	0.012
1	42/99	9.75 (7.83-11.67)		
2	74/109	6.00 (4.94-7.06)		
3	6/7	5.75 (0.59-10.91)		

Abbreviations: CI, confidence interval; PFS, progression free survival; ECOG, Eastern Cooperative Oncology Group; DCP, des-gamma-carboxy prothrombin; Hb, hemoglobin; ALBI, albumin-bilirubin.

**p* value by univariate analysis

** *p* value by multivariate analysis

Table S8. Univariate and multivariate analysis of factors associated with progression-free survival (PFS) in the high-risk population.

Subgroup	Events/ patients	Median PFS, months (95% CI)	*p*-Value *	*p*-Value **
All patients	67/98	6.50 (3.93-9.08)		
Age, years ≤65	42/60	5.75 (2.86-8.64)	0.411	
>65	25/38	7.25 (2.53-11.97)		
Sex			0.624	
Male	55/84	7.00 (4.56-9.44)		
Female	12/14	3.25 (2.33-4.17)		
Etiology			0.957	
Hepatitis B	43/58	5.75 (3.38-8.12)		
Hepatitis C	4/10	18.50 (-)		
Hepatitis B + hepatitis C	1/1	1.25 (-)		
Non-viral	19/29	8.25 (3.42-13.08)		
ECOG performance status			0.198	
0	38/59	7.75 (5.32-10.18)		
1	29/39	5.00 (3.01-6.99)		
Child-Pugh score			0.066	
5A	32/53	8.25 (6.49-10.01)		
6A	19/26	4.75 (2.63-6.87)		
7B	13/15	3.00 (2.05-3.95)		
8B	3/3	9 (3.80-14.20)		
9B	0/1	-		
Ascites			0.223	
Absent at baseline	54/82	7.00 (4.81-9.19)		
Present at baseline	13/16	4.00 (2.21-5.79)		

Barcelona Clinic Liver Cancer stage			0.017	0.538
B	4/4	2.25 (0-4.70)		
C	63/94	7.00 (4.62-9.38)		
Alpha-fetoprotein at baseline (ng/ml)			0.102	
≤400	29/46	9.00 (7.92-10.08)		
>400	38/52	4.75 (2.49-7.01)		
DCP at baseline (mAU/ml)			0.015	0.032
≤2000	21/39	9.25 (8.59-9.91)		
>2000	44/56	4.75 (2.96-6.54)		
Macrovascular invasion at baseline			0.020	0.211
No	14/17	2.75 (1.74-3.76)		
Yes	53/81	7.75 (5.75-9.75)		
Extrahepatic metastasis			0.460	
No	28/43	8.25 (5.29-11.21)		
Yes	39/55	5.00 (5.06-7.94)		
Vp4 portal vein thrombus			0.373	
No				
Yes	21/28	3.00 (1.70-4.30)		
	46/70	7.75 (5.71-9.79)		
Bile duct invasion			0.406	
No	49/75	6.50 (3.79-9.21)		
Yes	18/22	4.75 (2.01-7.49)		
Liver infiltration over 50%			0.006	0.400

No	28/50	9.00 (7.89-10.11)		
Yes	39/48	3.00 (2.36-3.64)		
Prior local therapy			0.797	
No	37/57	5.25 (2.85-7.65)		
Yes	30/41	8.25 (4.38-12.12)		
Neoadjuvant or concomitant radiation therapy			<0.001	0.018
No				
Yes	30/37	3.25 (2.27-4.23)		
	37/61	9.25 (7.66-10.84)		
Varices			0.676	
absent at baseline	25/39	4.00 (1.34-6.66)		
present at baseline	42/59	7.75 (5.14-10.36)		
Hb level (g/dL)			0.032	0.090
≤12.5	40/50	4.00 (1.85-6.15)		
>12.5	27/48	9.00 (6.46-11.54)		
Neutrophil to lymphocyte ratio			0.029	0.145
≤2.25	12/22	10.00 (8.11-11.89)		
>2.25	55/75	4.75 (2.49-7.01)		
ALBI grade			0.002	0.009
1	14/27	9.25 (6.68-11.82)		
2	49/67	4.75 (2.83-6.67)		
3	4/4	1.00 (0.00-5.90)		

Abbreviations: CI, confidence interval; PFS, progression free survival; ECOG, Eastern Cooperative Oncology Group; DCP, des-gamma-carboxy prothrombin; Hb, hemoglobin; ALBI, albumin-bilirubin.

*p value by univariate analysis

** p value by multivariate analysis

Table S9. Profiles of adverse events in the total population.

	Any grade, *n* (%)	Grade 5	Grade 3-4	Grade 1-2
Proteinuria	51 (23.7)	0	4	47
Aminotransferase elevation	36 (16.7)	0	1	35
Thrombocytopenia	35 (16.3)	0	0	35
Neutropenia	23 (10.7)	0	0	23
Hypertension	16 (7.4)	0	0	16
Azotemia	14 (6.5)	0	2	12
Subclinical hypothyroidism	13 (6.0)	0	0	13
General weakness	13 (6.0)	0	3	10
Dermatitis	13 (6.0)	0	2	11
Diarrhea	11 (5.1)	0	2	9
Hyponatremia	10 (4.7)	0	0	10
Poor oral intake	9 (4.2)	0	2	7
Varix bleeding	9 (4.2)	0	9	0
Epigastric pain	8 (3.7)	0	0	8
Pyrexia	8 (3.7)	0	1	7
Hypokalemia	7 (3.3)	0	1	6
Jaundice	7 (3.3)	0	2	5
Back pain	6 (2.8)	0	0	6
Nausea	6 (2.8)	0	2	4
Abdominal pain	6 (2.8)	0	0	6
Adrenal insufficiency	6 (2.7)	0	1	5
Pneumonitis	6 (2.7)	2	2	2
Hepatic encephalopathy	5 (2.3)	0	4	1
Anemia	5 (2.3)	0	1	4
Pancreatitis	4 (1.9)	0	0	4

Headache	4 (1.8)	0	0	4
Peripheral edema	4 (1.8)	0	1	3
Neutropenia	3 (1.4)	0	1	2
Ascites	3 (1.4)	0	1	2
Gastric ulcer	3 (1.4)	0	3	0
Ulcer bleeding	3 (1.4)	0	3	0
Dizziness	3 (1.4)	0	0	3
Stomatitis	3 (1.4)	0	0	3
Hyperkalemia	3 (1.4)	0	1	2
Constipation	2 (1.0)	0	0	2
Hemoptysis	2 (1.0)	0	2	0
Chest discomfort	2 (1.0)	0	1	1
Knee pain	2 (1.0)	0	0	2
Tingling sensation	2 (1.0)	0	0	2
Hypercalcemia	2 (1.0)	0	0	2
Cough	2 (1.0)	0	0	2
Hematuria	2 (1.0)	0	1	1
Hyperuricemia	2 (1.0)	0	0	2
Arm pain	1 (0.5)	0	0	1
Autoimmune hepatitis	1 (0.5)	0	1	0
Esophageal ulcer	1 (0.5)	0	1	0
Septic shock due to cholangitis	1 (0.5)	0	1	0
Pneumothorax	1 (0.5)	0	1	0
Spontaneous bacterial peritonitis	1 (0.5)	0	1	0
Hemobilia	1 (0.5)	0	1	0
Fracture	1 (0.5)	0	1	0
Bowel necrosis	1 (0.5)	0	1	0

Nocturia	1 (0.5)	0	0	1
Hand foot syndrome	1 (0.5)	0	0	1
Hypogeusia	1 (0.5)	0	0	1
Periodontitis	1 (0.5)	0	0	1
Right inguinal pain	1 (0.5)	0	0	1
Nose bleeding	1 (0.5)	0	0	1
Perianal fistula	1 (0.5)	0	1	0
Duodenitis	1 (0.5)	0	0	1
Vomiting	1 (0.5)	0	0	1
Right leg pain	1 (0.5)	0	0	1
Weight loss	1 (0.5)	0	0	1
Brain mass	1 (0.5)	0	1	0
Hypersensitivity vasculitis	1 (0.5)	0	0	1
Nausea	1 (0.5)	0	0	1
Nasal discharge	1 (0.5)	0	0	1
Vesicle	1 (0.5)	0	0	1
Toothache	1 (0.5)	0	0	1
Ureter stone	1 (0.5)	0	1	0
Duodenal perforation	1 (0.5)	1	0	0
Limb weakness	1 (0.5)	0	1	0

Figure S1. The mechanism of atezolizumab plus bevacizumab and radiation therapy combined with atezolizumab plus bevacizumab against hepatocellular carcinoma. [a] blocks the interaction between PD-L1 and B7.1 on immune cells to enhance T-cell priming; restores anti-cancer immunity through reactivation of T-cell mediated tumour cell killing [53]. [b] Normalises tumour vasculature, increasing T-cell infiltration [54–58]; Decreases the activity of immunosuppressive cells (MDSCs and Tregs) [54, 55, 59–62]; Promotes DC maturation [54,63,64].

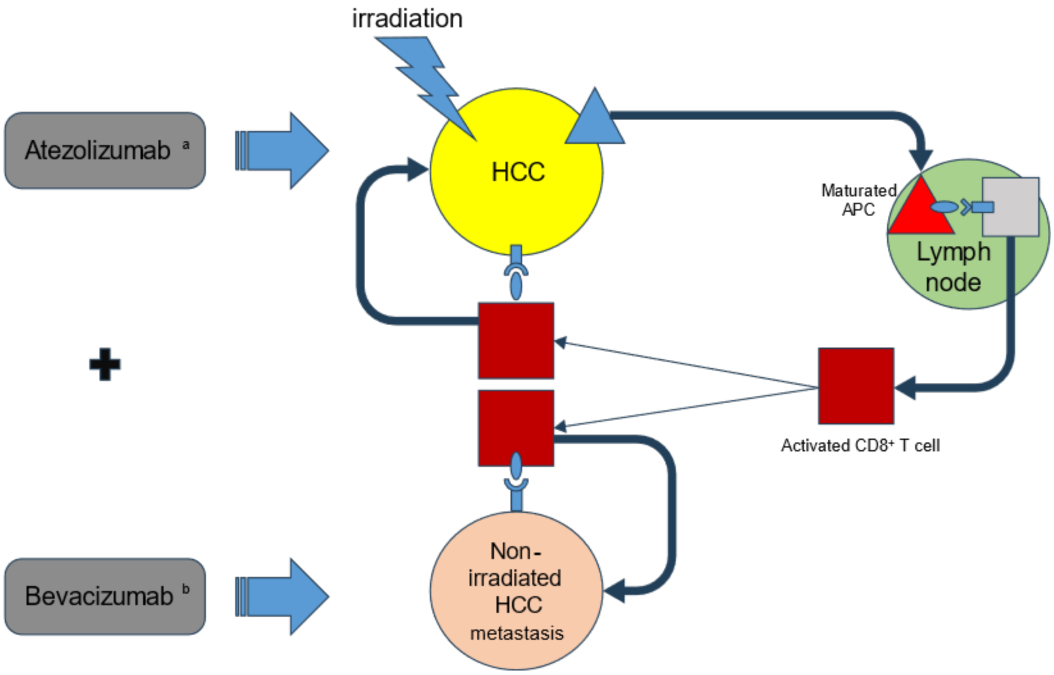

HCC, hepatocellular carcinoma; APC, antigen presenting cell; DC, dendritic cell.

Figure S2. Comprehensive flow sheet. In this study, ATE+BEV had consistent efficacy and tolerability in the total population and in the high-risk population. Receipt of neoadjuvant or concomitant radiation therapy might be helpful to improve the progression free survival and overall survival in the high-risk group.

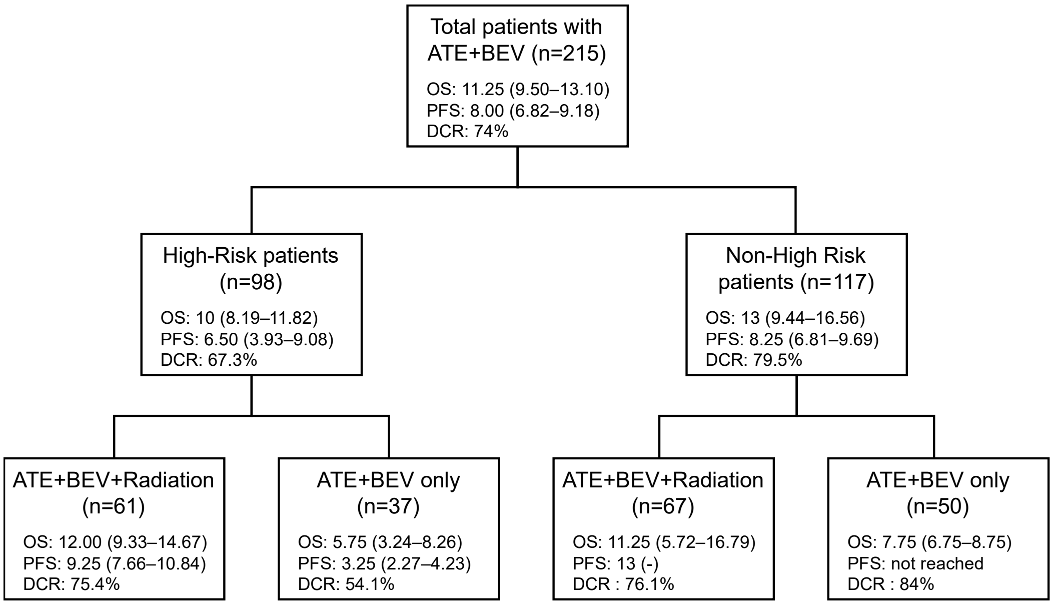

ATE, atezolizumab; BEV bevacizumab; OS, overall survival; PFS, progression free survival; DCR, disease control rate.

Figure S3. Infographic abstract

To evaluated the real-world efficacy and safety of atezolizumab plus bevacizumab in high risk (grade Vp4 portal vein thrombus, bile duct invasion, or more than 50% liver infiltration) patients with advanced hepatocellular carcinoma.

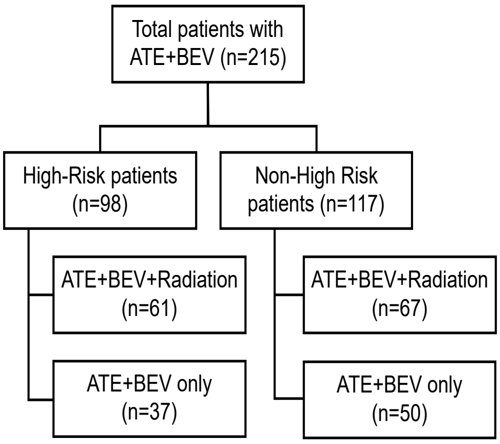

	OS (months)	PFS (months)
Overall group (N=215)	11.25 (9.50-13.10)	8.00 (6.82–9.18)
High-risk group (N=98)	10 (8.19-11.82)*	6.50 (3.93–9.08)#
with RT (N=61)	12.00 (9.33-14.67)§	9.25 (7.66–10.84)§
without RT (N=37)	5.75 (3.24-8.26)§	3.25 (2.27–4.23)§
Non-high risk group (N=117)	13 (9.44-16.56)*	8.25 (95% CI, 6.81–9.69)#

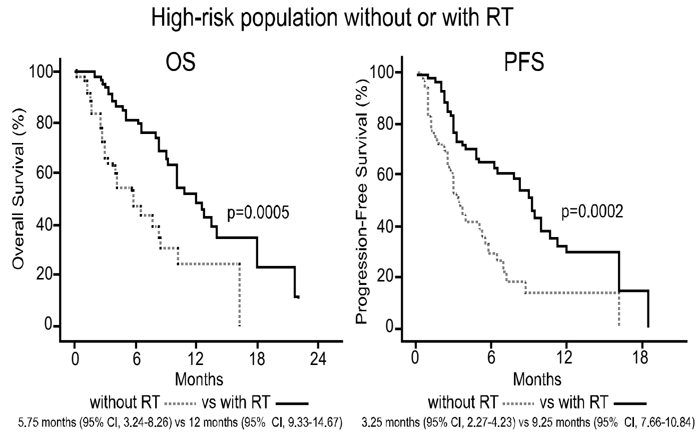

In this study, ATE+BEV had consistent efficacy and tolerability in the total population and in the high-risk population. Receipt of neoadjuvant or concomitant radiation therapy might be helpful to improve the progression free survival and overall survival in the high-risk group.

ATE, atezolizumab; BEV bevacizumab; OS, overall survival; PFS, progression free survival; RT, radiation therapy.

* *p*=0.004,

\# *p*=0.010

§ *p*<0.001

Figure S4. Comparative finding of pre and post treatment.

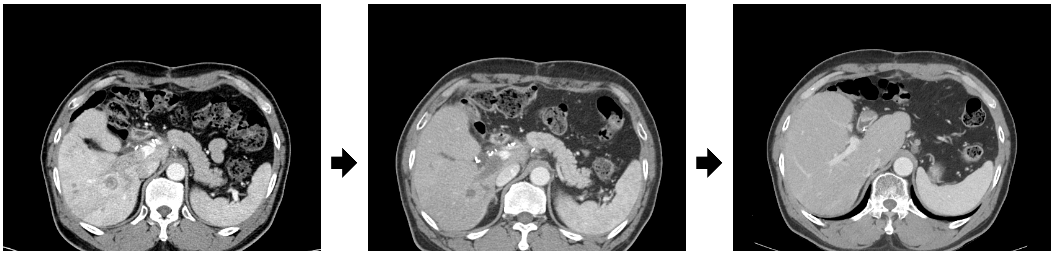

The patient was a 65-year-old male patient who had liver cirrhosis due to hepatitis B and was taking tenofovir. Pretreatment MRI and CT scans showed multiple hepatocellular carcinoma (HCC) and portal vein tumor thrombosis (PVTT) at the time of visit, so atezolizumab and bevacizumab was started. After 3 cycles of treatment, in the CT scan, the HCC became smaller, PVTT improved, and PIVKA2 decreased. After 3 more cycles of atezolizumab + bevacizumab, PVTT improved significantly in the CT scan, and there was no intrahepatic HCC and lymph node metastasis, and PIVKA2 decreased to normal values, indicating a complete response.

References

53. Chen, D.S.; Mellman, I. Oncology meets immunology: the cancer-immunity cycle. *Immun.* **2013**, *39*, 1–10. doi: 10.1016/j.immuni.2013.07.012.
54. Hedge, P.S.; Wallin, J.J.; Mancao, C. Predictive markers of anti-VEGF and emerging role of angiogenesis inhibitors as immunotherapeutics. *Semin. Cancer Biol.* **2018**, *52*, 117–124.
55. Wallin, J.J.; Bendell, J.C.; Funke, R.; Sznol, M.; Korski, K.; Jones, S.; Hernandez, G.; Mier, J.; He, X.; Hodi, F.S.; et al. Atezolizumab in combination with bevacizumab enhances antigen-specific T-cell migration in metastatic renal cell carcinoma. *Nat. Commun.* **2016**, *7*, 12624. doi: 10.1038/ncomms12624.
56. Goel, S.; Duda, D.G; Xu, L.; Munn, L.L.; Boucher, Y.; Fukumura, D.; Jain, R.K. Normalization of the vasculature for treatment of cancer and other diseases. *Physiol. Rev.* **2011**, *91*, 1071–121. doi: 10.1152/physrev.00038.2010.
57. Motz, G.T.; Santoro, S.P.; Wang, L.P.; Garrabrant, T.; Lastra, R.R.; Hagemann, I.S.; Lal, P.; Feldman, M.D.; Benecia, F.; Coukos, G. Tumor endothelium FasL establishes a selective immune barrier promoting tolerance in tumors. *Nat. Med.* **2014**, *20*, 607–15. doi: 10.1038/nm.3541.
58. Voron, T.; Colussi, O.; Marcheteau, E.; Pernot, S.; Nizard, M.; Pointet, A.L.; Latreche, S.; Bergaya, S.; Benhamouda, N.; Tonchot, C.; et al. VEGF-A modulates expression of inhibitory checkpoints on CD8+ T cells in tumors. *J. Exp. Med.* ***2015***, *212*, 139–48. doi: 10.1084/jem.20140559.
59. Gabrilovich, D.I.; Nagaraj, S. Myeloid-derived suppressor cells as regulators of the immune system. *Nat.Rev. Immunol.* **2009**, *9*, 162–74. doi: 10.1038/nri2506.
60. Roland, C.L.; Lynn, K.D.; Toombs, J.E.; Dineen, S.P.; Udugamasooriya, D.G.; Brekken, R.A. Cytokine levels correlate with immune cell infiltration after anti-VEGF therapy in preclinical mouse models of breast cancer. *PLoS One.* **2009**, *4*, e7669. doi: 10.1371/journal.pone.0007669.
61. Facciabene, A.; Peng, X.; Hagemann, I.S.; Balint, K.; Barchetti, A.; Wang, L.P.; Gimotty, P.A.; Gilks, C.B; Lal, P.; Zhang, L. et al. Tumour hypoxia promotes tolerance and angiogenesis via CCL28 and T(reg) cells. *Nature.* **2011**, *475*, 226–30. doi: 10.1038/nature10169.
62. Oyama, T.; Ran, S.; Ishida, T.; Nadaf, S.; Kerr, L.; Carbone, D.P.; Gabrilovich, D.I. Vascular endothelial growth factor affects dendritic cell maturation through the inhibition of nuclear factor-kappa B activation in hemopoietic progenitor cells. *J. Immunol.* **1998**, *160*, 1224–32.
63. Gabrilovich, D.I.; Chen, H.L.; Girgis, K.R.; Cunningham, H.T.; Meny, G.M.; Nadaf, S.; Kavanaugh, D.; Carbone, D.P. Production of vascular endothelial growth factor by human tumors inhibits the functional maturation of dendritic cells. *Nat. Med.* **1996**, *2*, 1096–103. doi: 10.1038/nm1096-1096.
64. Hodi, F.S.; Lawrence, D.; Lezcano, C.; Wu, X.; Zhou, J.; Sasada, T.; Zeng, W.; Giobbie-Hurder, A.; Atkins, M.B.; Ibrahim, N.; et al. Bevacizumab plus ipilimumab in patients with metastatic melanoma. *Cancer Immunol. Res.* **2014**, *2*, 63242. doi: 10.1158/2326-6066.CIR-14-0053.
